# The effect of an autologous cellular gel-matrix integrated implant system on wound healing

**DOI:** 10.1186/1479-5876-8-59

**Published:** 2010-06-17

**Authors:** Caroline R Weinstein-Oppenheimer, Alexis R Aceituno, Donald I Brown, Cristian Acevedo, Ricardo Ceriani, Miguel A Fuentes, Fernando Albornoz, Carlos F Henríquez-Roldán, Patricio Morales, Claudio Maclean, Sergio M Tapia, Manuel E Young

**Affiliations:** 1 Departamento de Bioquímica, Facultad de Farmacia, Universidad de Valparaíso, Avenida Gran Bretaña 1093, Playa Ancha Valparaíso, Casilla 5001-V, Valparaíso, Chile; 2 Departamento de Ciencias Farmacéuticas, Facultad de Farmacia, Universidad de Valparaíso, Avenida Gran Bretaña 1093, Playa Ancha Valparaíso, Casilla 5001-V, Valparaíso, Chile; 3 Departamento de Biología y Ciencias Ambientales, Facultad de Ciencias, Universidad de Valparaíso, Avenida Gran Bretaña 1111, Playa Ancha Valparaíso, Casilla 5030, Valparaíso, Chile; 4 Centro de Biotecnología "Daniel Alkalay", Universidad Técnica Federico Santa María, Casilla 110-V, Valparaíso, Chile; 5 Facultad de Ciencias Naturales y Exactas, Universidad de Playa Ancha de Ciencias de la Educación Avenida Leopoldo Carvallo 270, Playa Ancha, Valparaíso, Chile; 6 Centro de Estudios Estadísticos, Universidad de Valparaíso, Avenida Gran Bretaña 1041, Playa Ancha Valparaíso, Casilla 5030-V, Valparaíso, Chile; 7 Departamento de Estadística, Facultad de Ciencias, Universidad de Valparaíso, Avenida Gran Bretaña 1111, Playa Ancha, Valparaíso, Chile; 8 Clínica Veterinaria La Protectora, Levarte 833, Playa Ancha, Valparaíso, Chile; 9 Hospital Clínico IST, Alvarez 662, Viña del Mar, Chile

## Abstract

**Background:**

This manuscript reports the production and preclinical studies to examine the tolerance and efficacy of an autologous cellular gel-matrix integrated implant system (IIS) aimed to treat full-thickness skin lesions.

**Methods:**

The best concentration of fibrinogen and thrombin was experimentally determined by employing 28 formula ratios of thrombin and fibrinogen and checking clot formation and apparent stability. IIS was formed by integrating skin cells by means of the *in situ *gelification of fibrin into a porous crosslinked scaffold composed of chitosan, gelatin and hyaluronic acid. The *in vitro *cell proliferation within the IIS was examined by the MTT assay and PCNA expression. An experimental rabbit model consisting of six circular lesions was utilized to test each of the components of the IIS. Then, the IIS was utilized in an animal model to cover a 35% body surface full thickness lesion.

**Results:**

The preclinical assays in rabbits demonstrated that the IIS was well tolerated and also that IIS-treated rabbit with lesions of 35% of their body surface, exhibited a better survival rate (p = 0,06).

**Conclusion:**

IIS should be further studied as a new wound dressing which shows promising properties, being the most remarkable its good biological tolerance and cell growth promotion properties.

## Background

Natural polymers such as collagens, glycosaminoglycans, starch, chitin and chitosan have been used as biomaterials for skin substitutes because they closely resemble the native cellular milieu [1-3].

An interesting new matrix was proposed by Liu et al, 2004[4], that crosslinks chitosan, gelatin and hyaluronic acid, generating a mechanically resistant porous matrix able to support fibroblast growth. We choose this matrix as the basis to build a new system by including a fibrin gel.

Chitosan, a polysaccharide composed of glucosamine and N-acetyl glucosamine, obtained from N- deacetylation of chitin, is an excellent biomaterial due to its low cost, scale availability, anti-microbial activity and biocompatibility [5]. It has been used as a cross-linked scaffold for tissue engineering with polymers such as gelatin and hyaluronic acid, resulting in a biomaterial with improved biological and mechanical properties [6]. The use of fibrin in tissue engineering practices has been increasing over the last 10 years [7-11]. Fibrin is a gel formed by polymerization after the action of the enzyme thrombin. Even though it is not a part of the normal extracellular matrix, it is temporarily present during wound healing [12]. Its use as a polymeric support for the transplant of human skin has been reported showing improved proliferation, migration and differentiation of the cells compared to keratinocytes cultured in traditional cell culture flasks [3,8,13,14]. It was later reported that keratinocytes cultured on fibrin maintain the cells on a proliferative state and improves the take of the grafts containing these cells [4]. Hyaluronic acid, a glicosaminoglycan component of the connective tissue, is a linear polymer of d-glucuronic acid and N-acetyl-D-glucosamine [12]. Although a large amount of research has been focused on the use of the cross linked type of chitosan-based scaffolds for tissue constructs, the optimization of cell seeding is a critical step for the successful *in vitro *cultivation of artificial organs.

The aim of the present research was to investigate the performance of a novel artificial skin chitosan- based scaffold containing autologous skin cells that have been included in a fibrin gel integrated inside the matrix. The ultimate goal of this research was to explore the tolerance and the efficacy of an IIS on an animal-based model.

## Methods

### Biopsies

*Ortolagus cuniculus *rabbits were anesthetized with ketamine/xylasine (5 mg and 2 mg/100 g of body weight) [15]. A selected dorsal area was shaved and after disinfection with a povidone-iodine complex solution, a 1-cm^2^ biopsy was taken. All the animal experiments and procedures, including animal procurement, surgery, anesthesia, euthanasia, animal housing and surgery facilities were performed by veterinarians following the Facultad de Farmacia-Universidad de Valparaíso animal care guidelines, which are based on the Guide for the Care and Use of Laboratory Animals from the National Research Council[16].

### Cell isolation and culture

The technique used for isolation and culture of skin cells was adapted from previously published reports [17-19]. Briefly, the biopsy was washed three times with pH 7.4 0.1 M phosphate buffered saline (PBS) containing penicillin (100 U/mL)/streptomycin (100 μg/mL). Visible fat was mechanically removed and the remnant tissue was minced with surgical blades to optimize enzymatic digestion. Afterwards, epidermis was incubated with trypsine-EDTA (0.05%-0.53 mM) and dermis with collagenase (2 mg/mL). Dermal and epidermal cells were washed in DMEM and then fibroblasts were cultured in DMEM/F12 and keratinocytes in Defined Keratinocytes Medium. All the cell culture reagents were purchased from Invitrogen (Carisbad, CA, USA).

### Cell proliferation assay

The MTT (Sigma-Aldrich Co, St. Louis, MO, USA) assay, which has been validated as a proliferation assay even inside microcarriers [19-23], was used to determine cell proliferation within the IIS. Rabbit keratinocytes (13,000 cells) and fibroblasts (7,000 cells) growing in co-culture either on conventional cell culture flasks or in an IIS were utilized at passage 2 of primary cell culture. These cells were incubated with 0.5% MTT for 4 h at 37°C. Next, the scaffold was disaggregated with 0.5% trypsin-5.3 mM EDTA for 2 h (Invitrogen) at 37°C. Lysis buffer (3% w/v SDS and 40 mM HCl, in isopropanol) and ultrasound (15 minutes) were used to solubilize formazan. The resulting solution absorbance was read at 570 nm.

### Integrated Implant System (IIS) preparation

The procedure described by Liu et al [4], was followed to obtain a porous matrix. Briefly, a gelatin solution (1% w/v) is mixed, with a chitosan (2% w/v) solution, in 1% v/v acetic acid and hyaluronic acid (0,01% w/v) solution to form a polymeric scaffold, which was then cross linked by the use of 2- morpholine-ethane sulfonic acid (MES), 1-ethyl-(3,3-dimethyl-aminopropyl) carbodiimide (EDC) and N-hydroxysuccinimide (NHS). The cells were integrated onto the scaffold by *in situ *gelification of the fibrin (100 μL of both thrombin and fibrinogen per square centimeter).

The best concentration of fibrinogen and thrombin was experimentally determined by employing 28 formula ratios of thrombin and fibrinogen.

The optimal formula ratio (13 mg/mL fibrinogen and 130 NIH/mL thrombin plus 30 mM CaCl_2_) was selected to suspend a mixture of keratinocytes and fibroblasts. Fibroblasts and keratinocytes growing on separated T25 cell culture flasks (at passage 2 of primary cell culture) were tripsinized to recover both cell populations and seeded on the matrix to reach a final concentration of 3 × 10^4^ cell/cm^2 ^[24].

Afterwards, the IIS is incubated overnight until implanted. In order to evaluate the contribution of the cells in the healing process, the scaffold was also used as a cell free implant system (CFIS). Both systems had an average thickness of 3 mm and their shape and surface were tailored to the form of the skin lesion.

### Comparative preclinical assay

Six circular 2,5 cm diameter full-thickness excision wounds were performed at the paravertebral skin of eight young-adult rabbits. For each of the lesions, the following treatments were applied: IIS, CFIS (cell free integrated system), fibrin, autologous skin cells in fibrin, porous matrix or no treatment. The position of the treatment on the dorsal area of the rabbit was randomly assigned.

The performance of the treatments was evaluated by two blind referees, a medical doctor and a veterinarian. The outcome of each treatment was determined as graft take percentage, which is a clinical estimation of the area of the wound that is healed. Infection was categorized as a yes or no condition, and scar quality was scored based on color (1-5 scale), thickness (1-4 scale) and wound retraction (1-3 scale). The full description of the scale is summarized in Table 1.

**Table 1:** Treatment outcome evaluation

Variable	Units of measure
Graft take	Percentage

Infection	Yes/No

Scar color:	1 = hiperpigmentated 2 = non pigmented 3 = red 4 = almost normal 5 = normal

Scar thickness	1 = queloid 2 = hypertrophic 3 = almost normal 4 = normal

Scar retraction	1 = very retracted 2 = mild retraction 3 = no retraction

The blind referees utilized the above scale to evaluate the scar quality on the six wounds model of preclinical assay. The graft take percentage is the area of the wound that healed. When examining the presence of infection a yes was quantified as a 0, and a no was a 1. This scale gave an overall index for each wound on each rabbit.

### Preclinical efficacy assay

In order to evaluate the efficacy of an IIS, a 35% full thickness body surface lesion was performed on young adult rabbits. Twelve duplets of rabbits from the same progeny paired by body weight were either treated with an IIS or left with no treatment. The rabbits were assigned at random to each condition. The outcome of the treatment was determined by survival, weight gain and wound closure efficiency.

### Histological analysis

At the time of any wounds being clinically healed, treated or control, a biopsy of complete skin exceeding the initial size of the implant and including the region of wound healing, was taken. After fixing the biopsies in Bouin's solution for 24 h, they were rinsed in 70% ethanol, dehydrated until 95% ethanol, cleared in butanol, and embedded in Paraplast Plus (Sigma Chemical Co., St. Louis, MO, USA). Serial sections 5 μm thick were cut in a Leica RM 2155 microtome. A selected set of sections was mounted on albumin-coated microscope slides and stained with a trichromic stain (Sigma Chemical Co, USA). The sections were stained in Harris Hematoxylin for only 75 seconds, rinsed in running tap water for 10 min, and then rinsed in distilled water. Next, they were stained in 0.5% erythrosine B (C.I. 45430) - 0.5% orange G (C.I. 16230) for 30 min, and then rinsed in distilled water. They were then immersed for 10 min in 0.5% phosphotungstic acid and then rinsed in distilled water. Finally, they were stained in 1% methylene blue (C.I. 42780) for 75 seconds, and quickly dehydrated in 95% ethanol followed by 100% ethanol. After clearing in xylene, the slides were cover-slipped with Poly-Mount Xylene mounting medium (Polysciences, Inc., Warrington, U.S.A.). Afterwards, photomicrographs were taken in a Leitz-Leica DMRBE microscope equipped with a Nikon Coolpix 5000 digital camera.

For the PCNA immunochemical analysis, deparaffinized and rehydrated integrated implant system (IIS) sections were incubated for 5 min in a microwave oven (for antigen retrieval); then cooled down to room temperature, rinsed in distilled water and incubated in 3% H_2_O_2_ in absolute methanol (to block endogenous peroxidase activity). After rinsing in 50 mM 2-amino-2-(hydroxymethyl)propane-1,3-diol (tris), pH 7.6 buffer, the slides were incubated with 2% normal horse serum in the same buffer and later incubated overnight at 4°C in the monoclonal antibody to Proliferating Cell Nuclear Antigen (PCNA; 1/1000) (Zymed Laboratories Inc., CA, USA); the sections were subsequently incubated with biotinylated antimouse IgG (1/500) and then processed using peroxidase-ABC (standard kit, Vector Laboratories Inc. Burlingame, CA, USA) amplification procedure and DAB (Sigma Chemical Co.) as chromogen, and finally were slightly counterstained with Harris Hematoxylin for 10 seconds.

### Statistical procedures

#### Preclinical safety assay

Two variables were compared after 10 days post implant for the IIS and CFIS: percentage of graft take and scar color using the 1-5 ranking described in the above methods. Both variables are not normally distributed, therefore a standard nonparametric method was applied as described by Hollander and Wolfe [25].

#### Comparative preclinical assay

Pairs of rabbits were assigned at random to treatment with an IIS or to a control group. Success, which was assessed as the rabbit surviving, was compared between both groups, applying the Mc Nemar Test [25,26]. The null hypothesis was that the survival of the rabbits was identical in both groups. Twelve couples of rabbits were required to work with α value of 0.05 and a potency of 0.9. In addition, the variable area of cicatrisation was considered as an outcome for both the control and the case study rabbit. The area of cicatrisation was measured at the end of the analysis or at the last measurement recorded before the death of one of the pairs of rabbits. The Shapiro-Wilk test was utilized to check the normality assumption before applying the pairwise t test on the mean of area of cicatrisation.

## Results

### Fibrinogen-thrombin ratio

**Figure 1:**
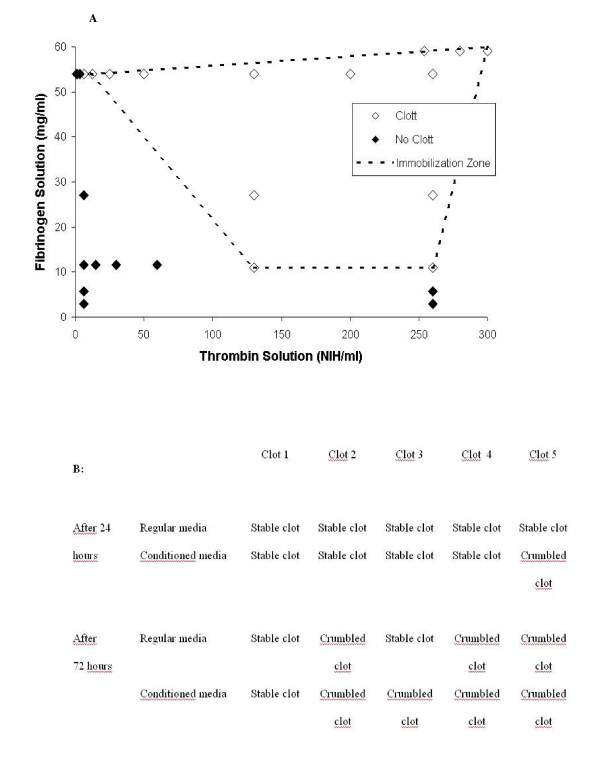
**Clotting characterization **50 uL fibrin clots were prepared with 25 uL of fibrinogen and 25 uL of thrombin at various concentrations of both. A: Clotting test on 28 fibrin formulations. Positive clotting was defined as the formation of a solid and homogenous clot. The dotted line shows the immobilization zone, where proper clotting was attained. B: Clot stability test for five selected formulas from the immobilization zone. The clots were cultured in regular cell culture media or in conditioned media in 24 well plates at 37°C. After 24 and 72 hours, clot samples were taken (n = 3) and visually examined to determine fibrin crumbling.

Twenty-eight different formulations changing fibrinogen and thrombin concentration ratio were evaluated for clotting formation and apparent stability as shown in Figure 1A. Clotting was obtained by mixing equal parts of fibrinogen and thrombin solutions, with a concentration range of between 3-60 mg/mL and 1-300 NIH/mL, respectively. The clot quality was assessed based on the capacity for clot formation, which is highly dependent on the cross linking that controls the mesh size of the network. Out of the 28 formula, 14 gave a clot, delimiting a feasible immobilization zone.

From the immobilization zone, five formulae were selected comprising a wide range of fibrinogen and thrombin concentration ratio. These five formulae were evaluated for stability, by incubating the clot in fresh cell culture media and in conditioned media, recovered from preconfluent skin cell cultures.

The optimal formula was chosen to be 13 mg/mL fibrinogen and 130 NIH/mL thrombin, which yielded fair clots that underwent fibrinolysis close after 24 h (Figure 1B), since it is desirable to obtain early fibrin degradation after implantation to allow cell delivery to the wound bed. The clotting time for this formula was approximately 1.2 sec.

### Cell proliferation within the IIS

**Figure 2:**
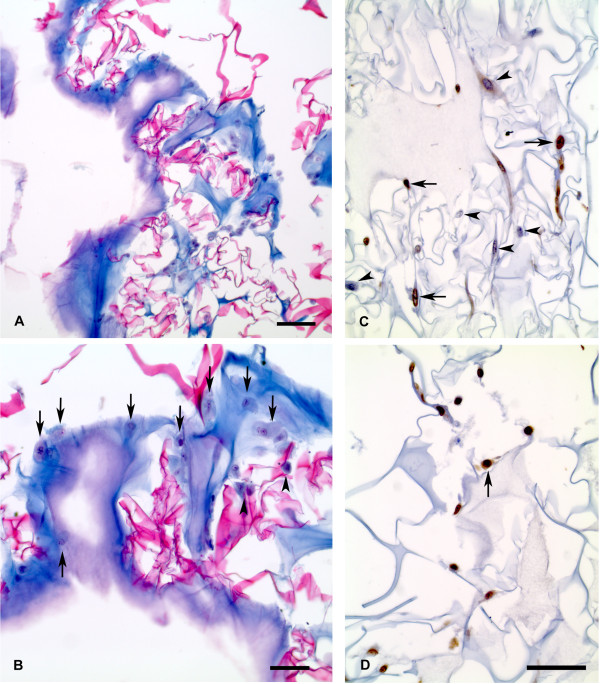
**Photomicrographs of histological sections from integrated implant system (IIS) stained with a trichromic stain (A-B) and immunohistochemical staining for Proliferating Cell Nuclear Antigen (PCNA) (C-D)**. A: The photomicrography at low magnification shows the fibrin in blue and the crosslinked scaffold in red. Scale bar = 100 μm (Panel A). B: At higher magnification, the skin cells embedded in the fibrin gel are indicated with arrows and with arrowheads when located within the cross-linked scaffold. Scale bar = 50 μm. C: Histological section showing the scaffold in pale blue, where the skin cells are immersed. Negative cells for anti PCNA antibody are shown with arrowheads, and positive nuclear reaction in cells is indicated with arrows. D: Another section of the scaffold is shown which exhibits only positive cells. Scale bar: C, D = 50 μm.

In Figure 2, a photomicrograph from the IIS after 24 hours of cell seeding is shown. Fibrin network stains with methyl blue, the scaffold appears as a red acidophilic fibrous net stained with erythrosine B (Figures 2A and 2B). It is important to highlight the close integration observed among all of the system components: matrix-gel-cells. At higher magnification, the presence of cells is shown in Figure 2B. The cells with their cytoplasm and nuclei stained in purple blue with the Harris Hematoxylin, are mainly located in the fibrin colloid (indicated by arrows) and also in the interphase with the reticular matrix (indicated by arrow heads).

**Figure 3:**
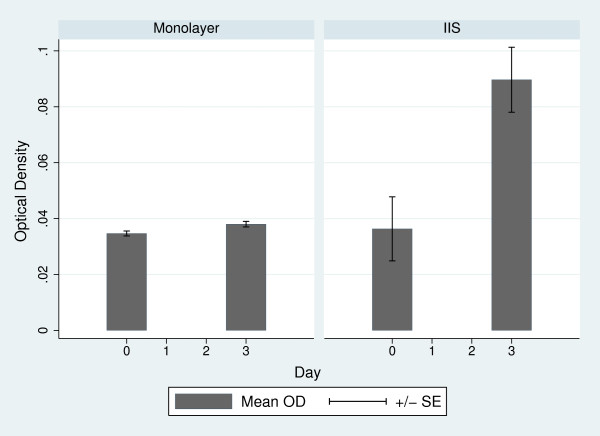
**Comparison of cell viability on monolayer versus IIS**. The MTT assay was performed at day 0 and after 72 hours of cell growth on conventional monolayer or in the IIS. To perform the MTT assay the scaffold was disaggregated by means of trypsine. The bars represent the average optical density of triplicates. Error bars are calculated as standard error.

The cell proliferation within the IIS was examined by the MTT assay and compared with cells grown in a conventional culture flask (Figure 3). After 72 hours of cell seeding, there was a noticeable increase of cell proliferation in the IIS compared to the cells grown in a conventional culture flask. In addition, histological sections of the IIS were immunohistochemically stained for Proliferating Cell Nuclear Antigen (PCNA) (Figures 2C and 2D), a marker of cellular proliferation. The microphotographs presented show cells positively stained for this antigen, further confirming that there are cells within the IIS which are proliferating.

### Comparative preclinical assay

Clinical evaluation in rabbits showed that after 10 days post implant, the graft take of CFIS was 53.75% and 81.25% for the IIS, demonstrating a significant statistical difference (p < 0.10). Evidence of mild infection was reported in 2 out of 8 rabbits for the CFIS, and also 2 out of 8 rabbits for the IIS. Scar color after 10 days was rated as 1.9, for the CFIS and 2.75 for the IIS, demonstrating a significant statistical difference (p < 0.05). There were no significant differences in the wound thickness or retraction of the scar during the evaluation period between the CFIS and IIS treated rabbits.

**Figure 4:**
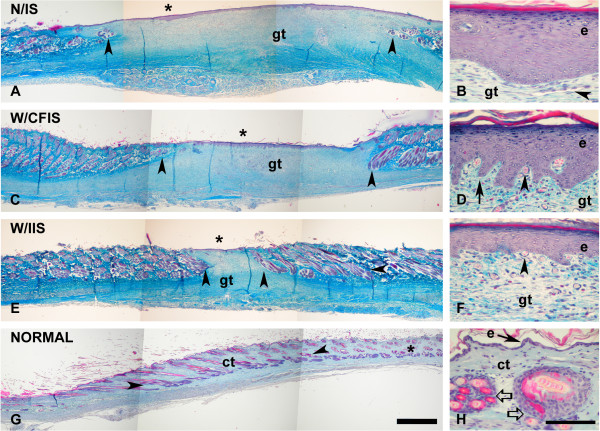
**Photomicrographs of histological sections from skin stained with a trichromic stain**. A: Wound healing in zone with no implant system (N/IS). Epidermis (asterisk) free of hairs and a region of granulation tissue below (gt), flanked by bundles of hair follicles (arrowheads). B High magnification on the asterisk region from A; thicker epidermis (e) with more than 15 nucleated cell layers and granulation tissue with blood vessels. C: Wound healing in zone treated with CFIS (W/CFIS) Epidermis (asterisk) free of hairs. Bulky granulation tissue (gt), flanked by packages of hair follicles (arrowheads). D: High magnification on the asterisk region from C; thicker epidermis (e) with more than 10 nucleated cell layers and basal finger like projections (arrow); granulation tissue with abundant blood vessels (small arrowhead). E: Wound healing in zone treated with IIS (W/IIS). Epidermis (asterisk) free of hairs and only a small region of granulation tissue below (gt), flanked by profuse bundles of hair.follicles (arrowheads). F: High magnification on the asterisk region from E; thicker epidermis (e) with more than 5 nucleated cell layers and granulation tissue with abundant blood vessels (small arrowhead). G: Normal skin (NORMAL) with very thin epidermis (asterisk) and below the connective tissue (ct) in pale blue, traversed by hair follicles (thick white arrow). H: High magnification on the asterisk region from G; epidermis (e) with no more than 2 nucleated cell layers and abundant connective tissue with bundles of hair follicles. Scale bar: A, C, E, G = 2 mm; B, D, F, H = 100 μm.

After 60 days, the wound surface was completely closed according to clinical evaluation, indicating that full epithelization occurred. A biopsy of the treated area was taken from each animal and processed as described in the above methods. Typical histological results are shown in Figure 4. Normal rabbit skin (Figure 4G) is characterized by a thin epidermis with no more than two nucleated cell layers and a dermis with connective tissue stained with methyl blue in a pale blue color and crossed with typical bundles of hair follicles (Figures 4G and 4H). In Figures 4C, D, E and 4F, treated wounds are presented with a complete epithelization, showing a thick epidermis and granulation tissue in the dermis, when compared with a normal skin biopsy (Figures 4G and 4H). In fact, in the treated wound, there is a zone of hair free epidermis, and the granulation tissue is also free of hair follicles (Figures 4C, D, E and 4F). The skin lesion treated with an IIS showed a tendency for smaller hair free areas (Figure 4E) than a CFIS treated lesion (Figure 4C) and than in untreated lesions (Figure 4A). Epidermis in the wound healed from the IIS treated lesion was thicker than the normal skin epidermis (around two nucleated cell layers, Figure 4H), although thinner (around 5 nucleated cell layers; Figure 4F ) than in a CFIS treated lesion, (around 10 nucleated cell layers; Figure 4D) and thinner than in the untreated lesion, (around 15 nucleated cell layers; Figure 4B). Overall, there was a better performance of an IIS in comparison to a CFIS.

It is important to highlight that at the time of the biopsy there were no signs of blood inflammatory cells like neutrophils, lymphocytes or macrophages, neither abscess or discharge of some kind of exudates such as serous, seropurulent, haemopurulent or pus, in any of the treated rabbits, typically associated with infection or rejection.

### Preclinical efficacy of the IIS

Rabbits which survived a 35% body surface lesion after an IIS treatment were compared with the untreated group utilizing the MacNemar test. This analysis indicated that the rabbits subjected to an IIS exhibited a better survival rate compared to the control group (p = 0.06).

The area of cicatrisation, of eleven pairs of rabbits was compared. One couple had to be withdrawn from this analysis because they died before five days of intervention.

The use of the Shapiro-Wilk procedure showed that the difference of area of cicatrisation is not rejected for the normality assumption required to use the paired t test (p > 0.55). This test indicated that the area of cicatrisation (open wound) of the IIS treated rabbits was significantly (p = 0.06) smaller than the control rabbits. In addition, a sub analysis was performed using just the pairs of rabbits in which neither the control nor the case rabbit died. In four pairs of rabbits, the control rabbits died before the end of the study (10, 15 and 30 days). With the seven pairs of rabbits that survived at least 50 days, the difference in the area of cicatrisation among ISS treated rabbits and control rabbits exhibited a higher statistical significance (p < 0.02).

**Figure 5:**
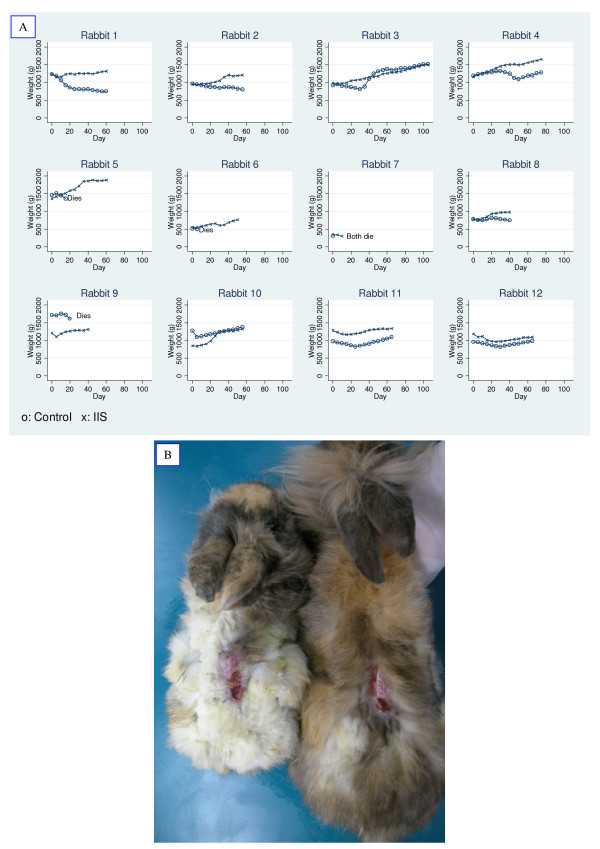
**Effect of an IIS treatment on the weight of rabbits undergoing a life threatening condition**. Panel A. Weight over time for each couple of rabbits. X: SII treated rabbit; 0 = untreated rabbit. Panel B. Representative picture of treated and non- treated animals after 60 days.

In addition, the majority of the rabbits in the IIS treated group showed a better curve of weight gain than the non treated animals (Figure 5A). The difference in growth, in some cases, was quite remarkable (Figure 5B).

## Discussion

This article describes a skin implant system built based on a novel approach that better integrates cells to a polymeric scaffold using fibrin as the cell carrier. The IIS was developed with the purpose of creating a wound dressing for regeneration of skin damaged by burns or other severe trauma. The IIS has the benefit of combining the presence of cells that has been reported by some authors as helping the healing process with fibrin which is a known natural component found in injured tissue at early stages of wound repair [3,27] and a scaffold which provides mechanical handling properties in addition to biological functionalities. The scaffold is composed of chitosan, which has been reported as an antibacterial agent [28], and an inductor of the formation of granulation tissue, angiogenesis, hemostasis and the production of interleukins which induce migration and proliferation of fibroblasts and keratinocytes [28-30]. The second component is hyaluronic acid, a major component of the extracellular matrix that has chemotactic and proangiogenic properties, in addition to being a scavenger of reactive oxygen species that are overall beneficial to the wound healing process [31]. The third component is gelatin, a low cost collagen-derived protein, which has been extensively used in several polymeric devices showing cytocompatibility, low immunoreactivity, adhesiveness, flexibility, promotion of cell adhesion and cell growth [1,2,32,33].

It is well known that the interaction between cells and the physical surface of cell culture play an important role in the outcome of the cells, influencing biological processes such as cell proliferation and differentiation. The developed system allows the cells to proliferate noticeably better than cells growing in conventional cell culture flasks. This might explain the positive clinical outcome of the IIS, since in a very short time after seeding, it improves cell proliferation within the system, a critical condition required to successfully treat severely injured skin. The observed high increase in cell proliferation may be the result of the cells actively secreting growth factors, such as PDGF [34], which may act either in a paracrine or autocrine fashion. It is known that fibrin chains (alpha and beta) and fibrinopeptides induce proliferation in skin cells by interacting with integrin receptors [35,36], and a partial degradation of fibrin stimulates fibroblast proliferation *in vitro *[37]. The ligation of integrin receptors in skin cells induces selective mRNA expression of many cytokines and growth factors such as PDGF-BB, EGF and TGF-β1 [38]. PDGF particularly stimulates fibroblast proliferation and the expression of integrin receptors [39]. In addition, it has been reported that cell adhesion to adequate substrates, results in a higher expression of cyclins of the G1 cell cycle phase [40]. Thus, the microenvironment within an IIS promotes cell activity which might result in the production of growth factors that are important for wound healing.

In agreement with other authors, the results presented, also point towards the beneficial effects of the presence of cells in the scaffold used for dermal restoration in the wound healing process [41,42]. Benefits are considered in terms of a better percentage of graft take (wound healing) and histological features such as epithelization, thickness of epithelia, and the area of granulation tissue, comparing the IIS with the CFIS, both referred to normal skin (Figures 4E and 4F with Figures 4C and 4D and Figures 4G and 4H, respectively). Furthermore, CFIS applied on its own, also showed a positive effect corroborated by the histological section taken from the lesion treated with it, showing thinner epithelia and a smaller area of granulation tissue than the control lesion (Figures 4C and 4D and Figures 4A and 4B, respectively).

The use of fibrin in dermal substitutes has shown several benefits like adjuvant of hemostasis and graft take. In addition, there is experimental evidence of fibrin acting as an antibacterial agent, a major challenge to the success of the dermal substitute. The mechanism of antibacterial action has been partially attributed to the stimulation of phagocytosis [3]. Despite the great benefits shown by several authors related to the use of fibrin as a delivery vehicle for skin cells, its weak mechanical properties have hampered its massive clinical use as a wound dressing. In this work, we present a device which allows for the use of fibrin as a cell vehicle but integrated in a scaffold, which provides mechanical strength, but also provides additional biological properties.

The preclinical experimental evidence supports that the IIS is well tolerated and efficacious because there were no signs of inflammation and all the wounds healed, showing complete epithelization. Moreover, when a life threatening lesion was performed, the IIS treated animals exhibited an overall better survival, better growth over time and smaller cicatrisation areas.

The use of autologous cells in this system is an advantage, not only because a scar of better quality is achieved, but also because it minimizes the infectious diseases transmission risk from one individual to another. However, the use of autologous cells might be seen as a drawback in view of the difficulties to store and transport of living cells and also higher costs due to their reduced possibilities of scale economy. Nonetheless, there is an autologous skin substitute currently available in the market, as well as, autologous dermal treatments for other applications. The use of animal-derived components, such as gelatine and fibrinogen, could be seen as a potential risk of transmission of certain animal borne infections, however, these and all of the components of IIS are available as pharmaceutical or tissue grade materials, presenting a risk which is comparable with products already within the pharmaceutical market.

The preclinical assays reported here show especially encouraging findings to continue with standardized clinical trials for the IIS and also to continue investigating the cell-biomaterial-skin interaction.

## Conclusions

An IIS is a wound dressing composed of known biomaterials combined in a novel approach, allowing the integration of the cellular component within the porous matrix. This gives the cells a microenvironment which promotes *in vitro *cell growth and constitutes a medical device that promotes wound healing at the preclinical level.

## List of abbreviations

**CFIS**: cell free implant system; **DMEM**: Dulbecco's Modified Eagle's Medium; **DMEM/F12**: Dulbecco's Modified Eagle's Medium/Ham's Nutrient Mixture F12; **EDC**: 1-ethyl-(3,3-dimethyl-aminopropyl) carbodiimide; **EDTA**: ethylenediaminetetraacetic acid; **IIS**: cellular gel-matrix integrated implant system; **MES**: 2-morpholine-ethane sulfonic acid; **MTT**: 3-(4,5-Dimethylthiazol-2-yl)-2,5-diphenyltetrazolium bromide; **NHS**: N-hydroxysuccinimide; **PBS**: pH 7.4 0.1 M phosphate buffered saline; **SDS**: sodium dodecyl sulfate.

## Competing interests

Young ME, Weinstein-Oppenheimer CR, Aceituno A, Acevedo C, Brown D and Tapia SM have a patent application for IIS.

## Authors' contributions

CWO: designed the scaffold, IIS and protocols for cell culture. She worked on the draft of the manuscript. ARA: designed and prepared the scaffold. He worked on the draft of the manuscript. DIB: performed the histological analysis and preclinical assay design and analysis. He worked on the draft of the manuscript. CA: designed cell viability assays and carried out fibrin/thrombin ratio experiments. He worked on the draft of the manuscript. RC: carried out the cell culture and cell viability assays. MAF: implemented the cell cultures and assembly of the IIS. FA: designed and prepared the IIS. CHR: performed statistical design and analysis of the preclinical assays. He worked on the draft of the manuscript. PM: was involved with the preclinical assays design, its performance and analysis. CM: participated in the design of the preclinical assay. SMT: participated in the design of the preclinical assay. MEY: developed the IIS and participated in the preclinical assay design. He worked on the draft of the manuscript.

All authors read and approved the final manuscript
